# Transcriptome Analysis of the Salt-Treated *Actinidia deliciosa* (A. Chev.) C. F. Liang and A. R. Ferguson Plantlets

**DOI:** 10.3390/cimb45050243

**Published:** 2023-04-27

**Authors:** Jiexin Wu, Zhuo Wei, Wenjuan Zhao, Zhiming Zhang, Daming Chen, Hanyao Zhang, Xiaozhen Liu

**Affiliations:** 1 Key Laboratory for Forest Resources Conservation and Utilization in the Southwest Mountains of China, Ministry of Education, Southwest Forestry University, Kunming 650224, China; wujiexin0815@gmail.com (J.W.); wz@swfu.edu.cn (Z.W.);; 2 Research Institute of Agriculture Ecological in Hot Areas, Yunnan Academy of Agricultural Science, Yuanmou 651300, China

**Keywords:** *Actinidia deliciosa*, salt treatment, transcriptome analysis, RT-qPCR

## Abstract

The area of saline land in the world is quite large, and there is broad room for its development and usage. ‘Xuxiang’ is an *Actinidia deliciosa* variety that is tolerant to salt and can be planted in an area of light-saline land, and has good comprehensive characteristics and high economic value. However, the molecular mechanism of salt tolerance is unknown at present. To understand the molecular mechanism of salt tolerance, the leaves of *A. deliciosa* ‘Xuxiang’ were used as explants to establish a sterile tissue culture system, and plantlets were obtained using this system. One percent concentration (*w*/*v*) of sodium chloride (NaCl) was employed to treat the young plantlets cultured in Murashige and Skoog (MS) medium, then RNA-seq was used for transcriptome analysis. The results showed that the genes related to salt stress in the phenylpropanoid biosynthesis pathway and the anabolism of trehalose and maltose pathways were up-regulated; however, those genes in the plant hormone signal transduction and metabolic pathways of starch, sucrose, glucose, and fructose were down-regulated after salt treatment. The expression levels of ten genes that were up-regulated and down-regulated in these pathways were confirmed by real-time quantitative polymerase chain reaction (RT-qPCR) analysis. The salt tolerance of *A. deliciosa* might be related to the expression level changes in the genes in the pathways of plant hormone signal transduction, phenylpropanoid biosynthesis, and starch, sucrose, glucose, and fructose metabolism. The increased expression levels of the genes encoding alpha-trehalose-phosphate synthase, trehalose-phosphatase, alpha-amylase, beta-amylase, feruloyl-CoA 6-hydroxylase, ferulate 5-hydroxylase, and coniferyl-alcohol glucosyl transferase might be vital to the salt stress response of the young *A. deliciosa* plants.

## 1. Introduction

At present, more than 100 countries are affected by soil salinization [1]. There are at least 100,000 hm^2^ of saline–alkali lands, of which 20% of the cultivated land is salinized [1]. Salt stress is one of the adverse environmental factors that causes plant growth inhibition and yield reduction [2]. Nowadays, the control of saline soil is mainly realized by improving saline soil, e.g., irrigation, organic fertilizer, deep tillage, loosening soil, and cultivating salt-tolerant plant varieties [1].

Cross-breeding to breed salt-tolerant varieties has become a vital topic in the academic research of salt-tolerant plant breeding [3]. However, because of the long breeding cycle of kiwifruit, the genetic improvement process of cross-breeding is seriously hindered [4,5]. A combination of traditional breeding and modern breeding techniques such as marker-assisted selection (MAS) is the trend in fruit tree breeding [4,5,6]. Hence, finding genes or molecular markers related to the traits is vital for fruit tree breeding. Modern techniques, for example, transcriptome, proteomics, and genomics can help screen salt-tolerance-related genes or molecular markers [2,7,8]. Combining cross-breeding with transcriptome sequencing technology to screen kiwifruit salt-tolerance-related genes is helpful for assisting in the breeding of salt-tolerant varieties [8,9,10].

Recent studies have shown that kiwifruit, like other salt-intolerant species, exhibit slowed growth, yellowing of leaves, and even death under salt-stressed environmental conditions [11]. The study of tree transcriptomes enables us to obtain new insights into the genetic basis for the formation of tree traits [7,8,10]. The study of salt stress response in plants has become a hot topic, and transcriptomic analysis has identified metabolic pathways associated with salt stress, including plant hormone signal transduction [12], lignin synthesis [13], starch, and the sucrose metabolism pathway [14,15]. 

The area of saline land is quite large, and there is broad room for its development and usage. *Actinidia deliciosa* ‘Xuxiang’ is one of the best kiwifruit varieties, which has good comprehensive characteristics and high economic value and can grow well in the light-saline land area [11,16]. However, the molecular mechanism of its salt tolerance is unknown. An in-depth study of the molecular regulation mechanism of salt tolerance and screening of salt-tolerance-related genes or molecular markers is essential to accelerate the breeding of kiwifruit varieties that can be planted in wilder saline land. In this study, to understand the molecular mechanism of salt tolerance and to screen the salt-tolerance-related genes of *A. deliciosa*, 1.0% (*w*/*v*) NaCl was used to treat the young plants, then expression levels of the salt tolerance-related genes were analyzed at the transcriptome level and confirmed by RT-qPCR analysis. 

## 2. Materials and Methods

### 2.1. Plant Materials and Tissue Culture

In this study, leaves of *A. deliciosa* ‘Xuxiang’ from the nursery of the Southwest Forestry University were used as explants for tissue culture.

The leaves of *A. deliciosa* ‘Xuxiang’ were inoculated in a callus induction medium (Murashige and Skoog (MS) + thidiazuron (TDZ) 1.0 mg/L + indole-3-butytric acid (IBA) 0.15 mg/L) for 20 days. An aseptic differentiation medium (MS + 6-benzyl aminopurine (6-BA) 1.0 mg/L+ naphthalene acetic acid (NAA) 0.2 mg/L) was used to induce the adventitious buds. They were moved to a rooting medium (MS+IBA 0.75 mg/L+ activated carbon (AC) 0.30 g/L) after they grew to 2 to 3 cm in height. The plantlets were grown in 15 h of light: 9 h of dark cycles at 25 ± 2 °C, with a light intensity of 1200 lx. After the roots grew to 2 to 3 cm, the seedlings moved to a greenhouse. 

### 2.2. Tissue-Culture Material Treatment

Three plants from tissue culture were placed in the salt medium with 1.0% (*w*/*v*) NaCl as reported by a previous study [11], numbered T1, T2, T3, and three plants were placed in the salt-free medium and numbered C1, C2, and C3. Seven days later [11], the leaves of the plants were removed, treated with liquid nitrogen, and placed in dry ice. These samples were sent to Differential Gene Company (Hefei, China) for RNA-Seq. 

### 2.3. Sequencing Data Processing

RNA was extracted using the Tiangen kit (TIANGEN Biotech Beijing Co., Ltd., Beijing, China) from one gram of each sample. The RNA quality was tested using a Nanodrop 2000 spectrophotometer (Thermo Fisher Scientific, Waltham, MA, USA). Using 5 μg RNA, the cDNA library was generated according to the operation manual of the NEBNext^®^ Ultra™ RNA Library Prep Kit for Illumina^®^ (NEB, Ipswich, MA, USA). The transcripts were sequenced by Differential Gene Company (Hefei, China) using Illumina HiSeqTM 2500, and RNA-Seq data were presented at the Genome Sequence Archive of the BIG Data Center (accession number PRJCA002128). After the clean reads were obtained, the Bowtie2 v2.2.5 (https://bowtie-bio.sourceforge.net/bowtie2/index.shtml, accessed on 22 July 2022) was used to compare clean reads to *A. chinensis* ‘Hong Yang’ v2 genome (http://bioinfo.bti.cornell.edu/cgi-bin/kiwi/download.cgi, accessed on 21 July 2022) [17]. The DEseq2 software was used to analyze the differentially expressed genes (DEGs) [18]. The p-values were calculated and then corrected by the false discovery rate (FDR) [19,20]. The genes with a threshold of FDR < 0.05, log2FC (fold change for a gene) > 1, or log2FC < −1 were selected as the final DEGs.

### 2.4. Enrichment Analysis of Differentially Expressed Genes in the GO Database

After the differential genes were screened out, the distribution of these genes in GO (http://www.geneontology.org/, accessed on 24 July 2022) was studied, and the embodiment of sample differences in gene function was clarified. The number of genes per term was calculated, and hypergeometric tests were applied to identify metabolic pathways where differential genes were significantly enriched relative to all annotated genes. The GO term with FDR ≤ 0.05 was selected as the significantly enriched GO entry.

### 2.5. Kyoto Encyclopedia of Genes and Genomes (KEGG) Analysis of DEGs

The differential genes were mapped to each term of KEGG (http://www.genome.jp/kegg/, accessed on 25 July 2022). The pathway significance enrichment analysis was based on the KEGG pathway and the hypergeometric test, and the number of genes per term was calculated using R software v.4.0.3. The metabolic pathway with FDR ≤ 0.05 was defined as a DEG that was enriched significantly.

### 2.6. Real-Time Quantitative Polymerase Chain Reaction (RT-qPCR) Analysis

Four genes in the pathway of phenylpropanoid biosynthesis and six genes in the starch and sucrose metabolism were selected for checking using RT-qPCR in 1% (*w*/*v*) NaCl-treated and control plantlets. The total RNA was extracted from the plantlets in the sequencing data processing section, and RT-qPCR analysis was conducted according to the method described by Li et al. [21]. The primers listed in Table 1 were for RT-qPCR analyses.

### 2.7. Determination of the Growth of A. deliciosa ‘Xuxiang’ Plantlets after Salt Treatment 

The plantlets grew well in a medium containing 0.5 % (*w*/*v*) NaCl, while growth in a medium with 1.0 % (*w*/*v*) NaCl was somewhat inhibited, but grew well. After weighing the flasks containing differentiation medium proliferation of 0.5% (*w*/*v*) NaCl and without NaCl, the plantlets of the same age (one month old) were inserted and the initial plantlet weight X was obtained. After 30 days, the operation was repeated, and the fresh weight Y of *A. deliciosa* ‘Xuxiang’ plantlets after 30 days of growth was obtained. The value of Y-X is the growing weight of *A. deliciosa* ‘Xuxiang’ plantlets within 30 days. Five biological replicates were set.

### 2.8. Data Statistical Analysis

The correlation coefficient between RNA-seq and RT-qPCR data was calculated using Excel 2007.

## 3. Results

### 3.1. Morphology of Salt-Treated Plants

**Figure 1:**
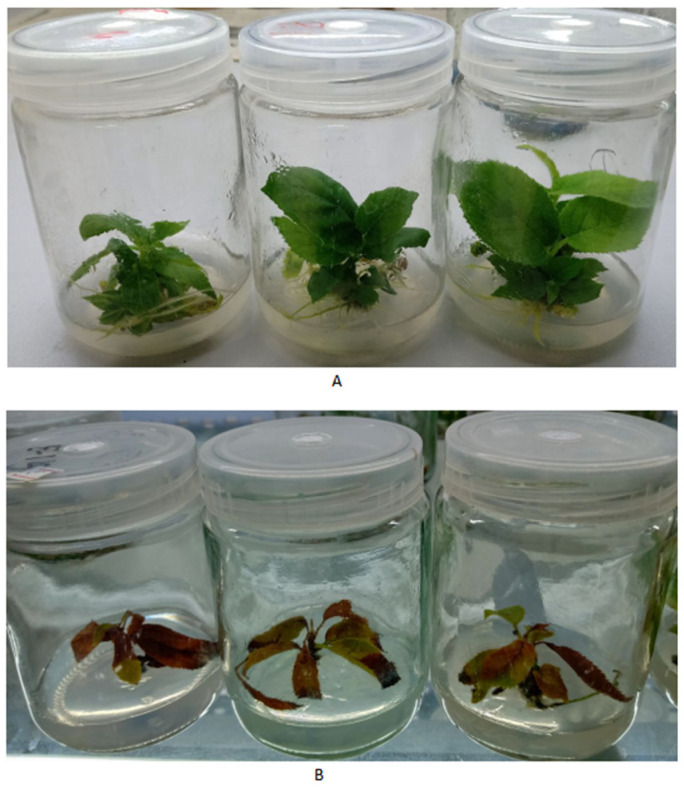
Control and salt-treated plants grew in a medium containing 1.0% (*w*/*v*) NaCl for 30 days. (**A**) Control plants; (**B**) salt-treated plants.

**Figure 2:**
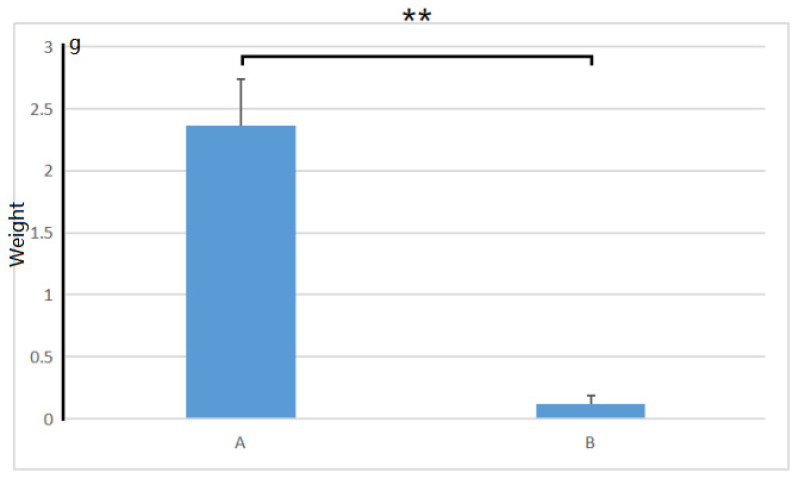
The growth weight of *A. deliciosa* ‘Xuxiang’ seedlings in 0.5% (*w*/*v*) NaCl within 30 days. A, Plants in the salt-free medium; B, plants in the salt-contained medium. **, *p* < 0.01.

After being treated with 1.0% (*w*/*v*) NaCl for 30 days, we found that the growth condition of control plants was significantly better than that of the salt-treated plants. The control plants could grow very well in the salt-free medium. Meanwhile, the leaves in the salt-contained medium turned yellow and withered, and the plants almost died (as shown in Figure 1). There was a significant difference in plant growth weight between salt-treated and non-salt-treated plants within 30 days (*p*-value = 0.000193, shown in Figure 2). After 30 days, the plants grown on the salt-containing medium did not emit any new shoots at the base, while the plants grown on the salt-free medium gave an average of 3.2 new shoots. The plants on the salt-containing medium had an average of 0.40 roots without root hair on the roots. The plants on the salt-free medium had an average of 8.2, and the main ones could grow lateral roots and root hairs.

### 3.2. The Quality of RNA-Seq

The proportion of nucleotides with quality values greater than the 20 (Q20) rate and proportion of nucleotides with quality values greater than the 30 (Q30) rate of all samples were more than 94%, and the contents of GC were between 40% and 50%. The proportion of unknown bases was 0, and the rRNA content was 0.1%, much lower than 10% of the indices (see Table 2). This indicated that the quality of the sequencing results was good. 

In six samples, the genes with the lowest expression levels (fragments per kilobase of exon model per million mapped fragments, FPKM 0~3) were 32–36%; the genes with low expression levels (FPKM 3~15) were 32–38%; and the genes with middle expression levels (FPKM 15~60) were 20–23%. The genes with high expression levels (FPKM ≥ 60) were between 6% and 8% (see Table 3). 

### 3.3. DEGs Analysis

**Figure 3:**
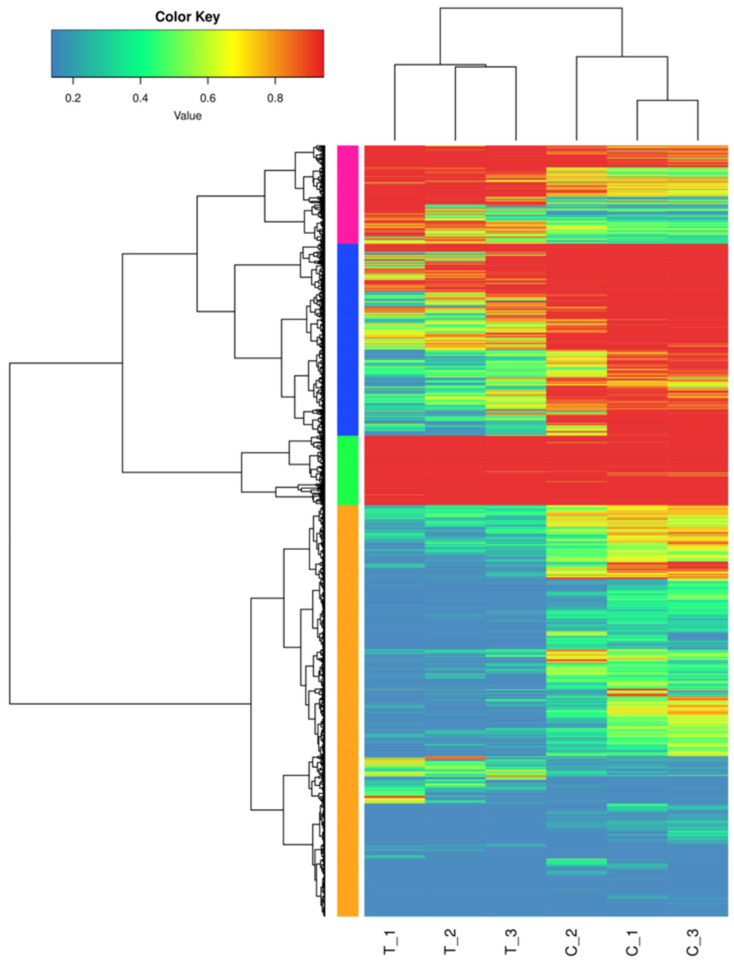
Cluster analysis of DEGs between salt-treated and control plants.

The results of the DEGs comparison between the treatment and control groups are shown in Table 4. The number of DEGs between the treatment and control groups was 5555, with 1546 genes up-regulated and 4009 genes down-regulated. Additionally, the total number of differential genes and up-regulated and down-regulated genes in the treatment group was significantly higher than that in the control group. Figure 3 shows differences in the transcriptome profiles between salt-treated and control plants.

**Figure 4:**
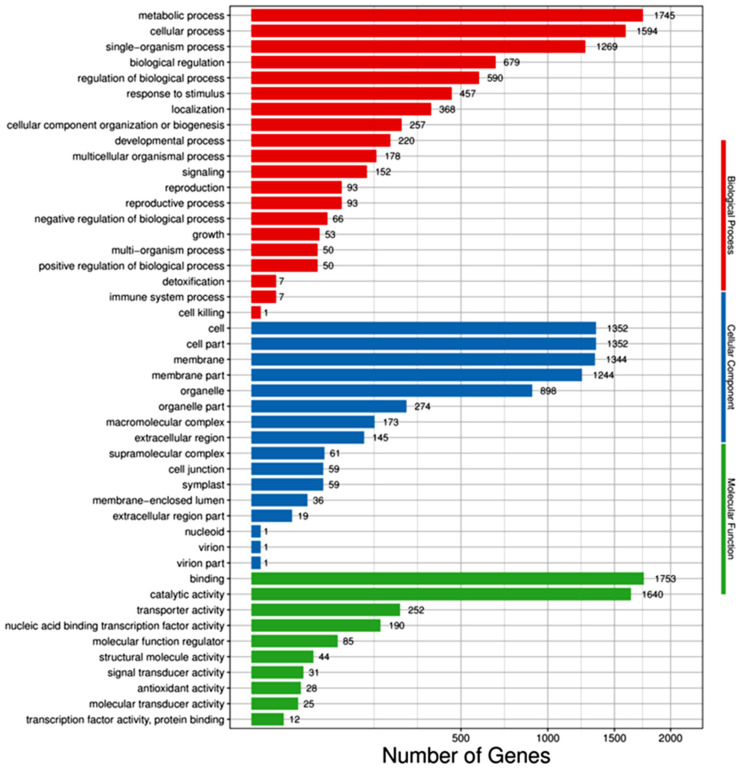
GO classification bar of DEGs between salt-treated and control plants.

The differential genes were annotated and assigned to the molecular function, biological process, and cellular components in the GO database. Ten functional classification items were enriched in molecular function, twenty in biological function, and sixteen in cell components (see Figure 4). Most of the differential genes were in the biological one. The metabolic, cellular, and single-organism processes, the biological regulation, and the regulation of the biological process were the top five items enriched, in which there were 1755, 1594, 1269, 679, and 590 differential genes, respectively. Second, there were also many differential genes in the cellular component, and the items enriched in the top five categories were cell, cell part, membrane, membrane part, and organelle, with 1352, 1352, 1344, 1244, and 898 differential genes, respectively. The least abundant of the differential gene was the molecular function. The items enriched in the top five were binding, catalytic activity, transporter activity, nucleic acid binding transcription factor activity, and molecular function regulator, with 1755, 1640, 252, 190, and 85 differential genes, respectively. 

The enrichment of all differential genes was similar to that of down-regulated differential genes, and the enrichment of all differential genes was slightly different from that of the up-regulated differential genes. The up-regulated differential genes were enriched in the cellular components, and the top five items were membrane, membrane part, cell, cell part, and organelle. Therefore, it could be inferred that the genes related to stomatal regulation may be up-regulated after salt stress. The differential genes enriched in these entries might be closely related to salt tolerance.

### 3.4. KEGG Analysis of DEGs

The results showed that 1423 significantly different genes were annotated in the KEGG pathway and enriched in 117 metabolic pathways. Among them, 420 up-regulated differential genes belonged to 82 metabolic pathways, and 1003 down-regulated differential genes belonged to 114 metabolic pathways. The top 15 pathways with the accumulation of differential genes are shown in Table 5. After filtering by Q-value (Q < 0.05), only seven were left. Among them, the enrichment of the top five pathways included plant hormone signal transduction, phenylpropane biosynthesis, starch and sucrose metabolism, the transformation of pentose and glucuronic acid, and the MAPK signaling pathway. The pathway of plant hormone signal transduction (ko04075) enriched 126 differential genes, of which 25 were up-regulated and 101 were down-regulated. The phenylpropane biosynthesis pathway (ko00940) enriched 70 differential genes, including 30 up-regulated and 40 down-regulated genes. The starch and sucrose metabolism pathway (ko00500) enriched 57 differential genes, including 17 up-regulated and 40 down-regulated genes. The transformation of the pentose and glucuronic acid pathway (ko00040) enriched 41 differential genes, consisting of 11 up-regulated and 30 down-regulated genes. The MAPK signaling pathway (ko04016) enriched 40 differential genes consisting of 21 up-regulated and 19 down-regulated genes.

**Figure 5:**
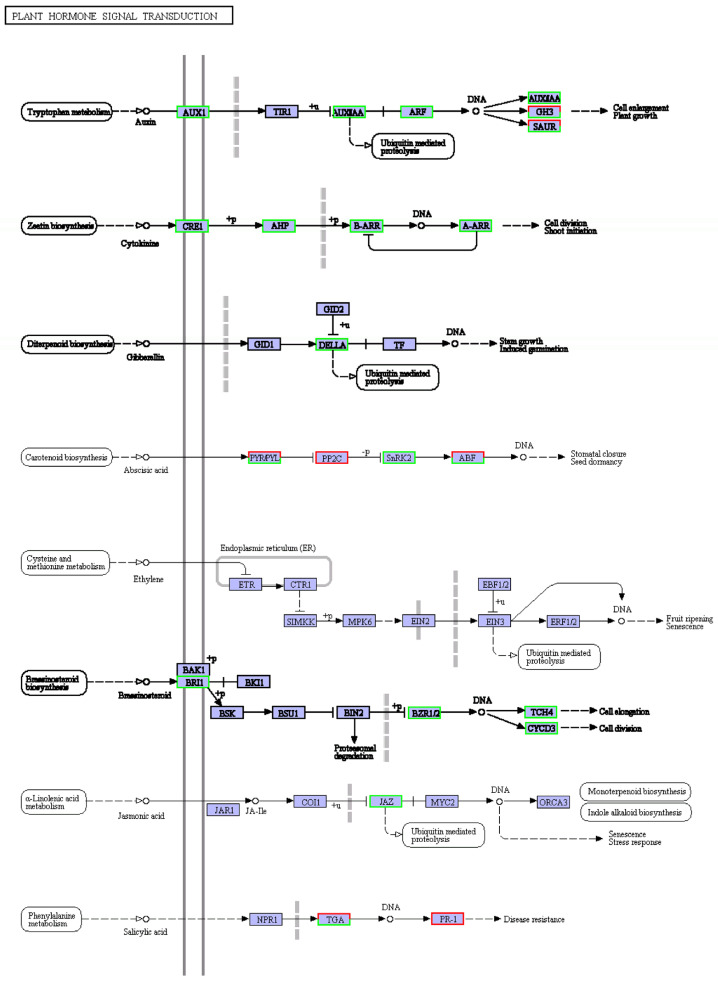
KEGG diagram of plant hormone signal transduction pathway under salt stress. Red box, up-regulated genes; green box, down-regulated genes; black box, no change in expression level compared to control. White in the box, no expression detected; blue, expression detected.

Plant hormones are involved in almost all life processes in plants, and the response of plants to salt stress is no exception [22]. Many down-regulated differential genes were enriched in the plant hormone signal transduction pathway (see Figure 5), for example, the auxin, cytokinin, gibberellin, and brassinosteroid signaling pathway. This could partly explain the significant slowing of plant growth under salt stress mentioned earlier (Figure 2).

**Figure 6:**
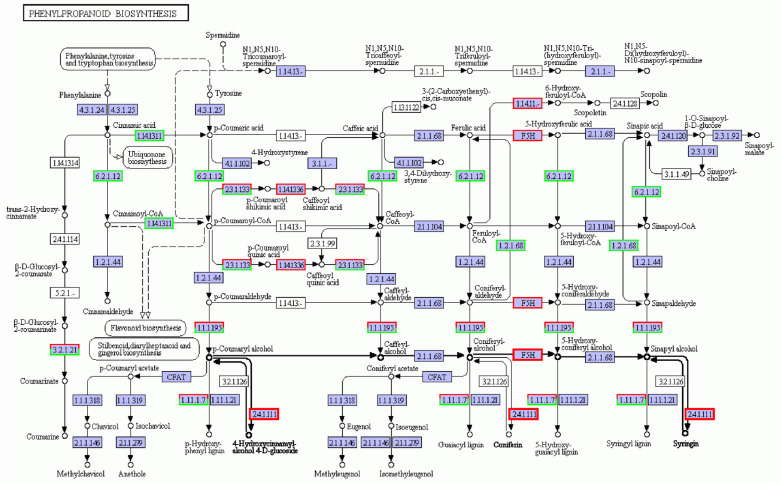
Phenylpropanoid biosynthesis metabolic pathway under salt stress conditions. Red box, up-regulated genes; green box, down-regulated genes; black box, no change in expression level compared to control. White in the box, no expression detected; blue, expression detected.

Phenylpropanes are ubiquitous in plants, and these secondary metabolites are characterized by hydroxyl aromatic rings [23]. There are thousands of chemical structures, including total flavonoids, flavonols, coumarins, lignin, anthocyanins, tannins, and other benzene compounds. These compounds play vital roles in plant growth, development, and stress response [24]. The phenylpropane biosynthesis pathway is shown in Figure 6. The biosynthesis pathway related to lignin synthesis responds to salt stress, and the regulatory network was complex. In this pathway, the number of down-regulated genes was more than that of the up-regulated genes, but the number of up- and down-regulation genes enriched in vital enzymes was similar, so it was hard to determine the positive and negative effects of its regulation. Among them, the genes encoding F5H and UGT72E2, the vital enzymes of lignin synthesis [25,26], were up-regulated. The gene encoding key enzyme F6H1, a tolerant coumarin compound, was up-regulated in salt-treated plants. 

**Figure 7:**
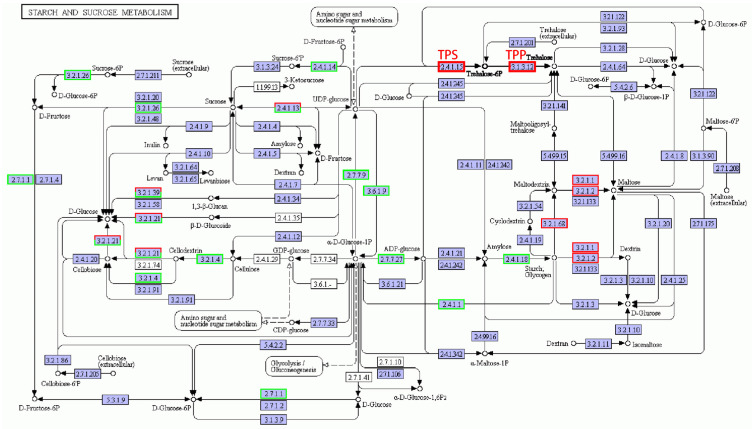
Starch and sucrose metabolic pathways under salt stress conditions. Red box, up-regulated genes; green box, down-regulated genes; black box, no change in expression level compared to control. White in the box, no expression detected; blue, expression detected.

The metabolic pathways related to starch and sucrose metabolites were very complex (as shown in Figure 7), and the number of down-regulated genes was more than that of the up-regulated ones. It may be because under salt, plants are affected by stress, and overall transcript levels are reduced, so most gene expression levels are decreased, but since starch and sucrose metabolites are vital for plant resistance, some key enzymes are essential for starch and sucrose synthesis, so the expression of these related coding genes was up-regulated. In this pathway, trehalose-6-phosphate synthase (TPS) and thiamine pyrophosphate (TPP), the key enzymes related to trehalose synthesis [27], responded to salt stress and were up-regulated. In the metabolic pathway of maltose synthesis, genes encoding α-amylase, β-amylase, and isoamylase were up-regulated. In the starch and sucrose metabolic pathway, genes related to the synthesis and metabolism of sucrose, starch, glucose, and fructose were down-regulated after salt stress, but the genes of trehalose and maltose synthesis were up-regulated.

### 3.5. RT-qPCR Validation

**Figure 8:**
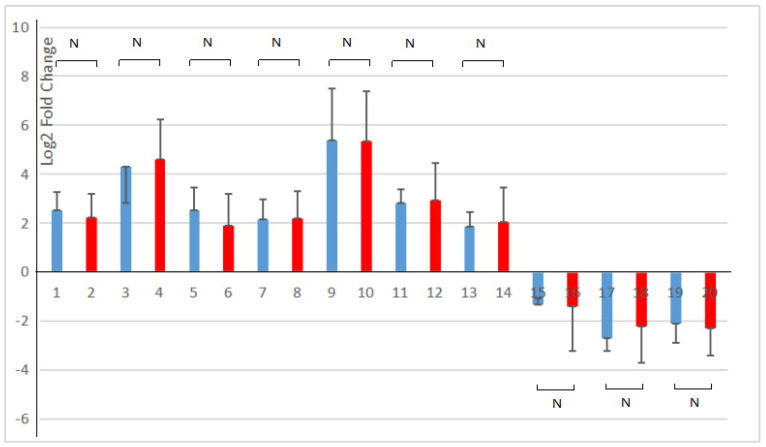
Comparison of RNA-sequencing and RT-qPCR results of the selected DEGs. Blue, RNA-seq; red, RT-qPCR. 1, 2. Alpha-trehalose-phosphate synthase gene; 3, 4. Trehalose-phosphatase gene; 5, 6. Alpha-amylase gene; 7, 8. Beta-amylase gene; 9, 10. Feruloyl-CoA 6-hydroxylase gene; 11, 12. Ferulate 5-hydroxylase (F5H) gene; 13, 14. Coniferyl-alcohol glucosyl transferase gene; 15, 16. 1,4-Alpha-glucan branching enzyme gene; 17, 18. UTP-glucose-1-phosphate uridylyltransferase gene; 19, 20. 4-Coumarate:CoA ligase gene. RT-qPCR was performed on three salt-treated (1% *w*/*v* NaCl) and three control seedlings, normalized with the housekeeping gene elongation factor. The 2^(−∆∆Ct)^ method was utilized to process the data [28]. N, *p* > 0.05.

All ten genes selected in the pathway of phenylpropanoid biosynthesis and starch and sucrose metabolism were consistent with the transcriptome analysis results (as shown in Figure 8). The correlation analysis showed that the expression levels of ten DEGs in RNA-seq and RT-qPCR had a strong positive correlation (R^2^ = 0.9858). It indicated that the results of the RT-qPCR analysis were in line with the transcriptome analysis after the young *A. deliciosa* ‘Xuxiang’ plants were treated with 1.0% salt. 

## 4. Discussion

Soil salinization or salt is one of the most crucial abiotic stresses that limits crop growth and productivity [29]. Various life activities of plants under salt stress are affected in varying degrees, such as inhibiting plant growth and development, destroying plant cell structure, and decreasing the synthesis of biomolecules needed to maintain plant physiological metabolism [30,31]. The way for glycophytes to avoid ion toxicity is to sacrifice part of the senescent tissue to protect the growth of plant seedlings [32]. The poor condition of plants caused by the high concentration of salt ions is not only characterized by physiological drought or difficulty in water absorption, but also due to the absorption of too much salt, which leads to the absorption of some nutrient elements that are beneficial to plant growth, thus inhibiting the growth and development of plants [33]. In this study, the leaves of salt-treated plants yellowed, plant growth stagnated, and even died, while the control plants grew very well. The views of Yang et al. partly confirmed our results [32]. 

As network regulation responds to abiotic stress, genes will up-regulate and down-regulate when the plants are treated with salt and other abiotic stress. Over-, under- or mixed expression of genes was found in abiotic stress-treated plants [13,34]. Amirbakhtiar et al. [29] found that 5128 genes were differentially expressed due to salt stress in the root of a salinity-tolerant wheat cultivar. Teshome et al. [35] identified 1555 transcripts of up-regulated genes and 1264 transcripts of down-regulated genes in response to salt stress in *Festulolium* hybrids. Jin et al. [36] compared transcriptomes from salt-treated and salt-free *Suaeda glauca* samples, and 231 DEGs were detected, including 130 up-regulated genes and 101 down-regulated genes. The comparative transcriptome analysis results of wheat roots under salt stress showed that there were 152 up-regulated genes and 5 significantly down-regulated genes [36]. In our study, 5555 DEGs were detected between the salt-treated and control group, with 1546 genes up-regulated and 4009 genes down-regulated. Some identified transcripts showed significant sequence similarity with genes to be up-regulated or down-regulated during salt and other abiotic stresses. The expression levels of the genes encoding alpha-trehalose-phosphate synthase, trehalose-phosphatase, alpha-amylase, beta-amylase, feruloyl-CoA 6-hydroxylase, F5H, and coniferyl-alcohol glucosyl transferase in salt-treated *A. deliciosa* ‘Xuxiang’ plantlets were significantly higher than those of control plants.

Among the plant hormone signaling pathways enriched in most differential genes, growth hormone, cytokinin, gibberellin, ethylene, and jasmonic acid metabolism were all down-regulated by salt stress, but salicylate metabolism was up-regulated (see Figure 5). The synthesis of growth hormone, cytokinin, gibberellin, and oleuropein lactone was reduced, especially the growth hormone synthesis pathway, which was enriched with many down-regulated differential genes. It suggests that when the ‘Xuxiang’ kiwifruit is subjected to salt stress, growth-related hormone pathways may be blocked, the synthesis of growth-promoting hormones is slowed down, and growth is inhibited. Noor et al. found that exogenous jasmonic acid can control salt stress [37]. However, the jasmonic acid-related genes were down-regulated, and the salicylic acid metabolism gene was up-regulated in the salt-treated ‘Xuxiang’ kiwifruit plants. Boamah et al. showed that salicylic acid could effectively protect the integrity of cell membranes during *Trichoderma longibrachiatum* seed germination and enhance the viability of its seeds under NaCl stress [38]. When ‘Xuxiang’ was subjected to salt stress, up-regulation of genes related to salicylate hormone synthesis led to a salt injury reduction and a salt tolerance increase.

Some transcripts are associated with the cell wall, in which lignin is the major component in salt tolerance [39]. The lignin content of plants was found to be positively related to salt stress [40]. Many genes from phenylpropanoid metabolism pathways that are known to take part in lignin biosynthesis were involved in the salt stress response [39,41]. Chun et al. [37] found that the lignin content of *Arabidopsis* plants increased after salt stress. In this study, the vital enzymes for lignin synthesis, F5H and UGT72E2, which are in the pathway for phenylpropane biosynthesis related to lignin synthesis, were up-regulated. We suggest that the up-regulation of key genes for lignin synthesis could provide more raw material for the synthesis of plant secondary cell walls, which would be more difficult to penetrate with salt when thickened. The up-regulation of key genes for lignin synthesis could thus serve as an indicator that the plant senses and responds to salt stress in the first place. 

Soluble sugar metabolism contributes to salt tolerance [13,14]. Starch and sucrose metabolism is involved in the salt stress response in plants [13,14,42,43]. For example, Chen et al. [43] found that DEGs associated with phenylpropanoid biosynthesis and starch and sucrose metabolism were involved in the salt stress response in maize. The genes in the sucrose, starch, glucose, and fructose synthesis and metabolism pathways were down-regulated after salt treatment, while the expression levels of the genes in the trehalose and maltose synthesis pathways were up-regulated. Sucrose, glucose, fructose, trehalose, and maltose are all soluble sugars [44,45], but only the expression levels of the genes in the trehalose and maltose synthesis pathways were up-regulated (see Figure 4). The accumulation of the soluble sugars trehalose and maltose was beneficial for improving the ability of plants to regulate osmotically and enhanced salt tolerance.

It has been pointed out that trehalose could form a unique protective layer on the cell surface to prevent the phase transition of the biofilm or replace the lost water membrane of biological macromolecules in harsh environments such as high osmotic pressure and effectively protect the structure of nucleic acids, proteins, and other macromolecules, and maintain the survival and biological characteristics of life [46,47]. Some TPS- and TPP-related genes are regulated in response to abiotic stresses, including salt stress in plants [48]. An increase in the content of trehalose can improve the salt resistance of plants. In this study, the expression levels of the differential genes encoding TPS or TPP were up-regulated after salt treatment.

## 5. Conclusions

The salt tolerance of *A. deliciosa* is related to the expression level changes in the genes in the pathways of plant hormone signal transduction, phenylpropanoid biosynthesis, and starch, sucrose, glucose, and fructose metabolism. The vital genes related to lignin synthesis were up-regulated, and the genes related to the synthesis and metabolism of sucrose, starch, glucose, and fructose were down-regulated after salt treatment, while the expression levels of the genes for the synthesis of trehalose and maltose were up-regulated. The expression levels of the genes encoding alpha-trehalose-phosphate synthase, trehalose-phosphatase, alpha-amylase, beta-amylase, feruloyl-CoA 6-hydroxylase, F5H, and coniferyl-alcohol glucosyl transferase were up-reregulated when the *A. deliciosa* ‘Xuxiang’ plantlets were treated with 1% salt. These genes might be vital to the salt stress response of the young *A. deliciosa* plants.

## Figures and Tables

**Table 1:** Primers of target genes (TG) and reference genes (RG) used in RT-qPCR.

Gene Name	Primer	Sequence (5′-3′)	Length (bp)
Elongation factor (RG)	Forward primer	ACAAGCTGGTGACAATGTGG	127
	Reverse primer	CGACCACCTTCATCCTTTGT	
Alpha-trehalose-phosphate synthase (CEY00_Acc20153, TG)	Forward primer	ATGACGGTACCATGATGCCT	168
	Reverse primer	GACCAAGCTTTTCACACGGT	
Trehalose-phosphatase (CEY00_Acc28912, TG)	Forward primer	GACAAGACCGATGAAGACGC	194
	Reverse primer	CTCCTCCTTGTTCGGGACTT	
Alpha-amylase (CEY00_Acc23514, TG)	Forward primer	ACTGGTGGATTGGGTAGTCG	198
	Reverse primer	TGCACCCTCGTTGTATTCCT	
Beta-amylase (CEY00_Acc00234, TG)	Forward primer	CTTGGAGATGGCGAAGAAGC	150
	Reverse primer	TCTGTGTAGGCAAGGTCAGG	
Feruloyl-CoA 6-hydroxylase (CEY00_Acc11474, TG)	Forward primer	GGTGATCAACGTCGGTGATG	222
	Reverse primer	AAGTGCCTGGTGTAATCCGA	
Ferulate 5-hydroxylase (MSTRG.9985, TG)	Forward primer	CGACGCATTTGAAGTAGGGG	224
	Reverse primer	ACATACAACCGGGCCGATAT	
Coniferyl-alcohol glucosyl transferase (CEY00_Acc06090, TG)	Forward primer	ATAAGGAGATCGCGGGTCAG	213
	Reverse primer	ACGCTTCCAGTGATTTACGC	
1,4-Alpha-glucan branching enzyme (CEY00_Acc23491, TG)	Forward primer	TGGTTGATCGAATCCCAGCT	181
	Reverse primer	GAGCTGCTCATCCCAACATG	
UTP-glucose-1-phosphate uridylyltransferase (CEY00_Acc24242, TG)	Forward primer	AGCACCACAGATTCCGAGAA	210
	Reverse primer	AGGATCTTCAGGTGTGGGTG	
4-Coumarate:CoA ligase (CEY00_Acc18560, TG)	Forward primer	ATGAGGCAGCAGGAGAAGTT	193
	Reverse primer	AGCTTCGCCCTCAGATCTTT	

**Table 2:** Quality evaluation of sample sequencing data after filtering.

Sample	Clean Reads	Base (M)	Q20 Rate	Q30 Rate	GC Rate	N Rate	rRNA Ratio
C_1	62,766,548	18,804	97.99	94.14	46.73	0	0.06
C_2	61,847,584	17,387	98.03	94.16	46.57	0	0.06
C_3	77,744,572	15,524	98.10	94.4	47.05	0	0.07
T_1	61,046,550	15,000	98.00	94.28	46.84	0	0.07
T_2	77,347,402	12,949	97.90	94.12	46.7	0	0.04
T_3	81,215,912	9656	97.07	92.04	46.61	0	0.04

**Table 3:** Statistics on the number of genes in different expression levels.

Sample	Expressed (Gene)	Total (Gene)	The Percent of Gene Expression			
			0~3 (Lowest)	3~15 (Low)	15~60 (Middle)	>60 (High)
C_1	34,641	36,355	12,468 (33.82%)	13,608 (37.43%)	8208 (22.58%)	2477 (6.81%)
C_2	34,487	36,355	11,889 (32.25%)	13,412 (36.89%)	8227 (22.63%)	2341 (6.44%)
C_3	34,438	36,355	11,526 (32.08%)	13,636 (37.51%)	7756 (21.33%)	2454 (6.75%)
T_1	33,787	36,355	13,017 (35.31%)	11,993 (32.99%)	7477 (20.57%)	2553 (7.02%)
T_2	33,791	36,355	13,303 (35.89%)	12,399 (34.11%)	8040 (22.12%)	2445 (6.73%)
T_3	33,469	36,355	13,014 (35.30%)	11,994 (32.99%)	8190 (22.53%)	2694 (7.41%)

**Table 4:** Statistics of gene differential expression analysis results.

Item	Total Number of DEGs	Number of Up-Regulated Genes	Number of Down-Regulated Genes
T-vs.-C	5555	1546	4009
T_1-vs.-C_1	3490	948	2542
T_2-vs.-C_2	1467	293	1174
T_3-vs.-C_3	1513	264	1249

**Table 5:** Top fifteen pathways of differential gene enrichment.

KEGG Pathway	Total Number of Genes	Number of Down-Regulated Genes	Number of Up-Regulated Genes	Q-Value
Plant hormone signal transduction (ko04075)	126	101	25	3.56 × 10^−17^
Phenylpropanoid biosynthesis (ko00940)	70	40	30	3.51 × 10^−12^
Starch and sucrose metabolism (ko00500)	57	40	17	4.56 × 10^−7^
Pentose and glucuronate interconversions (ko00040)	41	30	11	1.11 × 10^−7^
MAPK signaling pathway-plant (ko04016)	40	19	21	6.51 × 10^−2^
Plant–pathogen interaction (ko04626)	36	13	23	1.00 × 10^+00^
Cysteine and methionine metabolism (ko00270)	33	23	10	2.97 × 10^−2^
Phagosome (ko04145)	33	31	2	1.19 × 10^−1^
Aminosugar and nucleotide sugar metabolism (ko00520)	31	26	5	3.62 × 10^−1^
Biosynthesis of amino acids (ko01230)	30	25	5	1.00 × 10^+00^
Carbon metabolism (ko01200)	26	17	9	1.00 × 10^+00^
Glycerophospholipid metabolism (ko00564)	24	17	7	2.88 × 10^−1^
Pyrimidine metabolism (ko00240)	24	21	3	7.53 × 10^−1^
Flavonoid biosynthesis (ko00941)	23	12	11	1.96 × 10^−3^
Galactose metabolism (ko00052)	22	12	10	1.39 × 10^−2^

## Data Availability

All data generated or analyzed during this study are included in this published article. RNA-Seq data were presented at the Genome Sequence Archive of the Beijing Institute of Genomics (BIG) Data Center (accession number CRA002280, https://ngdc.cncb.ac.cn/search/?dbId=gsa&q=CRA002280, accessed on 18 July 2022).
